# SARS-CoV-2 neutralizing camelid heavy-chain-only antibodies as powerful tools for diagnostic and therapeutic applications

**DOI:** 10.3389/fimmu.2022.930975

**Published:** 2022-09-14

**Authors:** Anja Schlör, Stefan Hirschberg, Ghada Ben Amor, Toni Luise Meister, Prerna Arora, Stefan Pöhlmann, Markus Hoffmann, Stephanie Pfaender, Omar Kamal Eddin, Julian Kamhieh-Milz, Katja Hanack

**Affiliations:** ^1^ New/era/mabs GmbH, Potsdam, Germany; ^2^ Charité – Universitätsmedizin Berlin, corporate member of Freie Universität Berlin and Humboldt-Universität zu Berlin, Institute of Transfusion Medicine, Berlin, Germany; ^3^ Wimedko GmbH, Berlin, Germany; ^4^ Department for Molecular and Medical Virology, Ruhr-University Bochum, Bochum, Germany; ^5^ Infection Biology Unit, German Primate Center– Leibniz Institute for Primate Research, Göttingen, Germany; ^6^ Faculty of Biology and Psychology, University Göttingen, Göttingen, Germany; ^7^ Department of Biochemistry and Biology, University of Potsdam, Potsdam, Germany

**Keywords:** camelid heavy-chain-only antibodies, single domain antibodies, nanobodies, SARS-CoV-2, neutralization, Omicron

## Abstract

**Introduction:**

The ongoing COVID-19 pandemic situation caused by SARS-CoV-2 and variants of concern such as B.1.617.2 (Delta) and recently, B.1.1.529 (Omicron) is posing multiple challenges to humanity. The rapid evolution of the virus requires adaptation of diagnostic and therapeutic applications.

**Objectives:**

In this study, we describe camelid heavy-chain-only antibodies (hcAb) as useful tools for novel *in vitro* diagnostic assays and for therapeutic applications due to their neutralizing capacity.

**Methods:**

Five antibody candidates were selected out of a naïve camelid library by phage display and expressed as full length IgG2 antibodies. The antibodies were characterized by Western blot, enzyme-linked immunosorbent assays, surface plasmon resonance with regard to their specificity to the recombinant SARS-CoV-2 Spike protein and to SARS-CoV-2 virus-like particles. Neutralization assays were performed with authentic SARS-CoV-2 and pseudotyped viruses (wildtype and Omicron).

**Results:**

All antibodies efficiently detect recombinant SARS-CoV-2 Spike protein and SARS-CoV-2 virus-like particles in different ELISA setups. The best combination was shown with hcAb B10 as catcher antibody and HRP-conjugated hcAb A7.2 as the detection antibody. Further, four out of five antibodies potently neutralized authentic wildtype SARS-CoV-2 and particles pseudotyped with the SARS-CoV-2 Spike proteins of the wildtype and Omicron variant, sublineage BA.1 at concentrations between 0.1 and 0.35 ng/mL (ND50).

**Conclusion:**

Collectively, we report novel camelid hcAbs suitable for diagnostics and potential therapy.

## Graphical Abstract

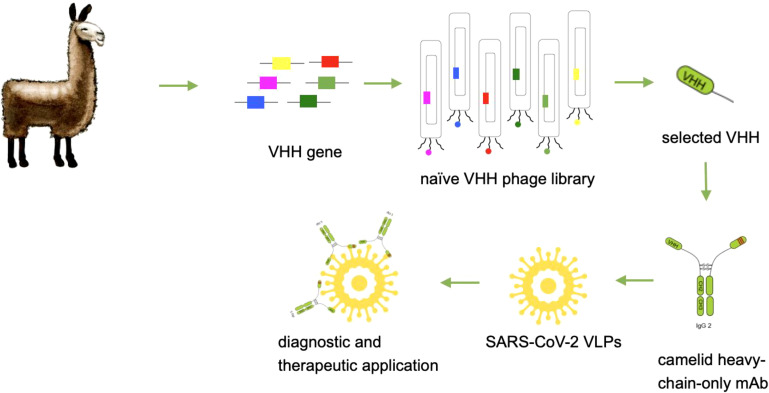

## Introduction

Camelid heavy-chain-only antibodies (hcAbs) are described as a novel class of binding molecules with highly promising advantages for diagnostic and therapeutic applications (1). Compared to mouse or human antibodies, hcAbs recognize their corresponding target only with one chain instead of a heavy and a light chain. The variable element of these heavy-chain-only antibodies is defined as camelid single domain antibody (VHH) or briefly as nanobody (2). Nanobodies are generated by phage display from naïve, immune or synthetic libraries and represent one of the smallest recognition unit in the class of antibody molecules with a molecular size of 15 kDa. They are characterized by a high thermal stability, a very good tissue penetration, and the recognition of hidden or difficult-to-reach epitopes (3). Therefore, these antibodies represent a new class of next-generation antibodies and are of high interest for biomedical applications. For *in vitro* diagnostic applications such as in ELISA systems, flow cytometry or immuno-fluorescence, nanobodies have to be expressed with a corresponding tag in order to provide a sufficient detection (4). Beside different tags, it can be advantageous to produce these nanobodies with a corresponding Fc part to create flexible opportunities for a sensitive detection and to allow a system which is compatible with standard procedures such as biotin/streptavidin or enzyme-linked reactions. In addition, the high thermostability which is a natural feature of camelid antibodies offers favourable opportunities for long-term storage, global distribution and manufacturability (5, 6). The generation and usage of camelid heavy-chain-only antibodies for *in vitro* diagnostic assays is therefore a promising approach. For therapeutic approaches it was published recently, that VHHs extended with Fc parts or as multimerized versions can increase their neutralization capacity, the serum half-life, and enhances effector functions (7–9). There are already several camelid antibodies and engineered formats described which neutralize SARS-CoV-2 (10, 11). Camelid antibodies have entered the antibody field and gain more and more attention due to their unique properties. As heavy-chain-only format they represent a new opportunity for the creation of novel *in vitro* diagnostic assay formats. Here, we describe the identification and recombinant production of camelid heavy-chain-only antibodies from a naïve camelid library that allow detection of SARS-CoV-2 with a high sensitivity and neutralize SARS-CoV-2 with a high potency.

## Material and methods

### Library construction and VHH-biopanning

A naïve camelid VHH library was generated as previously described by Schlör et al. (12). Briefly, RNA was isolated from peripheral blood mononuclear cells (PBMC; NucleoSpin RNA Mini Plus, Macherey-Nagel GmbH & Co.KG, Düren, Germany) from 3 different animals with 1-3x10^7^ cells. For cDNA first strand synthesis RevertAid kit (Thermo Scientific) was used with a combination of random hexamer and oligo dT primers. Following a 2-step PCR protocol for VHH amplification with the primer combination P12/13 and NEM01-03_PhD. In **Supplementary Figure S1** a scheme of the 2-step PCR principle is shown. Primer sequences are listed in **Table 1**. Amplified VHHs were cloned into phagemid vector pADL-22c (Antibody Design Labs). The library was transformed into *E. coli* XL1 blue (Agilent) by electroporation. After generation of the library, 55 clones were picked and checked with colony PCR for their corresponding VHH insert. Four clones showed no insert. Overall, 20 clones were sequenced and had their VHH insert in frame together with the gp3.

**Table 1:** Primer sequences for amplification of VHH sequences and cloning.

Primer name	Sequence	Description	Ref.
P12	GTC CTG GCT GCT CTT CTA CAA GG	Primers for first round of library amplification	(13)
	
NEM01_PhD	TCG CGG CCC AGC CGG CCA TGG CGC AGG TGC AGC TGC AGG AGT CTG GRG GAG G	Primers for second round of library amplification and cloning into pADL-22c (SfiI restriction sites)	(14)
NEM02_PhD	TGT TGG CCT CCC GGG CCG CTG GAG ACG GTG ACC TGG GT		
NEM02_PhD	TGT TGG CCT CCC GGG CCT GAG GAG ACG GTG ACC TGG GT		
SL13	TTC CAC TGG TGA TGT TCA GCT GCA GGA GTC TGG GGG	Primers for cloning VHH into pNEM_camAb using InFusion (Takara)	by ourselves
SL14	GTG TCT TGG GTT CGT TGC TGG AGA CGG TGA CCT GGG T		

For biopanning according to Barbas et al., transformed XL1 blue were cultured and inoculated with M13KO7 helper phages (15). The resulting phages were enriched in five rounds of biopanning. Full length SARS-CoV-2 Spike protein (SPN-C52H4; Acrobiosystems) was used as antigen in decreasing amounts starting from 15 to 0.1 µg. Post-panning analyses were performed as described by Coomber (16). Monoclonal phages were tested in phage enzyme-linked immunosorbent assays (ELISA) for antigen specific binding to recombinant SARS-CoV-2 Spike protein. Positive clones were sequenced and become heavy chain only antibodies by cloning into the expression plasmid pNEM_camAb.

### Heavy chain Ab design, expression and purification

For the generation of a heavy-chain-only antibody, the different VHHs were amplified with SL13/14 and cloned into the vector pNEM_camAb. pNEM_camAb was designed in our lab as vector for eukaryotic protein expression of camelid VHHs with a camelid IgG2b Fc fragment (GeneBank ID: AAX73259; IgG2b CH2-CH3 domain and hinge region) resulting in hcAbs.

HcAbs were expressed using the Expi293 expression system (Thermo Fisher Scientific). Culturing and transfection was performed according to the manufacturer’s protocol. After 5 to 6 days after transfection, the cell culture supernatants were harvested and filtered, followed by protein A purification (ProSep^®^ Ultra Plus; Merck). Elution of hcAbs was achieved by a pH shift with glycine buffer as described previously (17). Finally, the hcAbs were dialyzed against PBS (pH 7.4). The integrity and purity were analyzed using SDS PAGE, Western Blot and ELISA.

### Western blot analyses and ELISA

To investigate the different hcAb candidates in Western Blot, 1 µg SARS-CoV-2 Spike protein (antibodies-online, ABIN6952734) per lane was applied onto a 4-12% SDS polyacrylamide gradient gel. The gel was blotted using the NuPage system and and Bis-Tris buffer as transfer buffer. To block unspecific binding, Rotiblock was used as blocking agent and for the dilution of the first and the second antibody. Five different hcAb candidates were applied in a concentration of 1 µg/mL (diluted in Rotiblock) and incubated for 18 h at room temperature (RT). After washing the membrane, a horse-radish peroxidase (HRP) conjugated secondary antibody specific to camelid IgG2/3 (ABIN1981272, antibodies-online) was applied and incubated for 3 h at RT. WesternBright Sirius was applied as substrate solution. Images were taken after 45 sec to visualize the specific signal (A44241, Invitrogen, Carlsbad, CA, USA). As internal controls, human reconvalescent serum was used as a positive control (1:100) and His-tagged reference proteins (0.25 µg/mL) served as negative controls.

For the characterization of the binding different ELISA formats were used. Indirect ELISAs were performed by coating microtiter plates with 0.5 - 1 µg/mL full length SARS-CoV-2 Spike protein (SPN-C52H4; Acrobiosystems) in 1x PBS overnight at 4°C (50 µL/well), various hcAb concentrations (50 µL/well in PBS/1% casein or PBS/5% neonatal calf serum (NCS), 1 h incubation at RT) and 1-2 µg/mL of HRP-conjugated secondary antibody specific to camelid IgG2/3 (ABIN1981272, antibodies-online) in 50 µL/well supplemented with PBS/1% casein or PBS/5% NCS for 45 min incubation at RT.

For the phage ELISA, the wells were coated with full length SARS-CoV-2 Spike protein (3 µg/mL in PBS, 50 µL/well) and incubated with monophages (50 µL/well). Detection of the bound phages was done with HRP-conjugated anti-M13 (clone B62, produced in our lab) in a dilution of 1:8000. Sandwich ELISAs were performed with hcAbs in different concentrations on coated antigen as described above. Each ELISA was finished using TMB as substrate solution (50 µL/well, Carl Roth) and 1 M H_2_SO_4_ as stop solution. To block nonspecific protein binding, 1% casein in PBS or 5% NCS in PBS was used. Between the single steps the wells were washed 3 times with tap water and 10 times after the secondary antibody incubation. Optical density was measured at 450 nm with a 620 nm reference.

### HRP conjugation of hcAbs B10 and A7.2

HRP-conjugation of hcAbs was done using a modified version of the classical periodate method as published in 1985 (18). For activation of HRP, 0.5 mg/mL HRP in 100 mM NaHCO_3_ (pH 8.1) were mixed 1:1 with 12.5 mM NaIO_4_ and incubated at RT for 2 h in the dark. In the next step, the same volume of activated HRP and hcAbs (1 mg/mL in NaHCO_3_; pH 9.2) were incubated in a glass-wool plugged Pasteur pipet filled with Sephadex G-25 (GE Healthcare) for 3 h at RT in the dark. The elution of conjugated hcAbs from Sephadex was performed with 100 mM NaHCO_3_ (pH 9.2). To stop the reaction, 1/20th volume of 5 mg/mL NaBH_4_ was added and incubated at 4°C. After 30 min another volume (1/10) of freshly prepared NaBH_4_ solution was added and incubated at 4°C for 1 h. Finally, after an ammonium sulfate precipitation overnight HRP conjugated hcAbs were dissolved in PBS (pH 7.4).

### SPR analysis

The hcAb-binding properties of B10 were analyzed at 25°C on a Biacore T200 instrument (GE Healthcare) using 10 mM HEPES pH 7.4, 300 mM NaCl, 3 mM EDTA, 0.05% Tween 20, 0.25 mg/ml BSA as running buffer as previously described in (19). SARS-CoV-2 Spike protein-RBD-mFc (Acrobiosystem; SPD-C5259) was captured by a covalently immobilized anti-mouse IgG on a C1 sensor chip. Increasing concentrations of hcAb B10 (0.23 - 60 nM) were injected. Analyte responses were corrected for unspecific binding and buffer responses. The sensorgrams were fitted with a monovalente analyte model using Biacore evaluation software according to the instructions of the manufacturer (20). Since a Biacore SPR biosensor is a mass detector, the binding signal proportionally increases with increasing molecular weight. Knowing molecular weights of both interacting molecules and the immobilization level of the ligand, one can calculate the R_max theoretical_ (maximal binding signal of the analyte) assuming a 1:1 interaction and 100% active ligand according to the following formula:


$$\frac{\text{[RU}(\text{ligand immob}.)}{\text{/kDa aligand]}}\times \text{kDa analyte }={\text{R}}_{\text{max theoretical}}$$

By dividing the apparent Rmax determined by quantitative kinetic analysis with Rmax theoretical, percent ligand bound is calculated. Percent ligand bound can be referred to as stoichiometry of interaction or percent active ligands on the surface.

### Neutralization assay

To determine the neutralization capacity of hcAbs against SARS-CoV-2, a neutralization assay with a propagation-incompetent VSV*ΔG pseudovirus system or full length virus was performed as previously described (21). The expression vector for the Omicron spike (based on isolate hCoV19/Botswana/R40B58_BHP_3321001245/2021; GISAID Accession ID: EPI_ISL_6640919) was generated by Gibson assembly using five overlapping DNA strings (Thermo Fisher Scientific, sequences available upon request), linearized (BamHI/XbaI digest) pCG1 plasmid and GeneArt™ Gibson Assembly HiFi Master Mix (Thermo Fisher Scientific). Gibson assembly was performed according to manufacturer’s instructions. The pCG1 vector was kindly provided by Roberto Cattaneo (Mayo Clinic College of Medicine, Rochester, MN, USA). In specific, VSV*ΔG either bearing the SARS-CoV-2 (D614G) or SARS-CoV-2 B.1.1.529 (Omicron) Spike protein was incubated with a two-fold dilution of hcAbs and subsequently used to inoculated VeroE6 cells. Firefly luciferase (FLuc) activity was determined 18 hours post infection, and the reciprocal antibody dilution causing 50% inhibition of the luciferase reporter was calculated. In a similar experimental setup full length WT virus hCoV-19/Germany/BY-Bochum-1/2020 (B.1.1.70) (GISAID accession ID: EPI_ISL_1118929) (22) was incubated with a serial dilution of hcAbs and hereafter transferred onto VeroE6 cells. The cells were incubated for 72 h before they were stained with crystal violet to visualize cytopathic effects.

### Generation of SARS-CoV-2 virus-like particles

Human codon optimized sequences of genes encoding the S and E structural proteins of SARS-CoV-2 were synthesized by BioCat GmbH (Heidelberg, Germany) and subcloned into the pcDNA3.1 expression plasmid using the NheI 5’ and XhoI 3’ restriction site, respectively. The SARS-CoV-2 Spike protein contained the D614G mutation and the furin-cleavage site was destroyed (FKO) by R683A and R685A substitution. Another plasmid (pEXP-M+N) for the dual expression of the human codon optimized sequences of the M and N protein was generated by Vectorbuilder Inc. (VB200528-1033wpt). The two proteins were expressed from the same open reading frame separated by a T2A self-cleaving peptide and controlled by the human eukaryotic translation elongation factor 1 α1 promoter (EF1A). All plasmids were transformed into high efficiency chemically competent cells DH5α (New England BioLabs, Ipswich, MA, USA) using the heat shock method in a water bath at 42°C for 30 seconds, followed by shaking incubation in SOC outgrowth media at 37°C for 45 min. Next, 50 µL of the cell-containing media were plated on LB plates containing 50 µg/mL ampicillin. After incubation at 37°C overnight, resistant single colonies were picked and amplified in LB medium. The correct constitution of the plasmid was examined by restriction enzyme digest and the open reading frames were verified by DNA sequencing (Eurofins Genomics Germany GmbH, Ebersberg, Germany). Endotoxin free DNA was prepared using respective kits (Macherey-Nagel GmbH & Co. KG, Düren, Germany), quantified with a NanoDrop Spectrophotometer (Thermo Fisher Scientific, Wilmington, DE, USA), controlled by restriction digest and subsequently transfected into mammalian cells for VLP production. Expi293™ Expression System Kit, composed of Expi293 suspension adapted cell line, Expi293 transfection reagents and Expi293 culture medium was purchased from Thermo Fisher Scientific. Expi293 cells were grown at 37°C, 8% CO2 with 130-150 rpm on a Rotamax120 platform shaker (Heidolph Instruments GmbH & Co. KG, Schwabach, Germany) in Expi293 medium containing a final concentration of 100 U/mL penicillin-streptomycin. Cell diameter, the percentage of viable cells (vitality) and the concentration of viable cells were routinely monitored using LUNA Cell Counter (Logos Biosystems, Anyang, South Korea). Cells were seeded at a density between 0.3x10^6^ and 0.5x10^6^ cells/mL and subcultured when concentration reached 3x10^6^ to 5x10^6^ viable cells/mL which was typically after 3-5 days. For transfection, cells were precipitated for 5 minutes at 300 x g and subsequently resuspended in fresh media to a final concentration of approximately 3x10^6^ viable cells/mL and a vitality greater than 95%. Per 10^6^ cells approximately 1 µg of total DNA was transfected. The DNA mix was composed of pcDNA3.1-Spike (FKO), pcDNA3.1-E-Protein and pEXP-M+N-protein at a ratio of 6:2:3 diluted in Opti-MEM I Medium. To generate control VLPs without the Spike protein, the plasmid containing the Spike protein gene was replaced with a mock plasmid. ExpiFectamin 293 Reagent was also diluted in Opti-MEM I medium at the optimized ratio of 3.2 µL per 1 µg of DNA before it was slowly added to the DNA mixture. The ExpiFectamin DNA complex was incubated for 15 min at RT prior to the dropwise addition onto the gently agitated cells. Enhancers 1 and 2 were added approximately 20 h after transfection according to the manufactures protocol. Next, 96-120 h after transfection when the vitality usually decreased to 40-60%, cell culture supernatants where cleared by centrifugation at 2000 x g for 10 min followed by filtration with a 1.2 µm Minisart NML (Sartorius Stedim Biotech GmbH, Göttingen, Germany) and a 0.45 µm Millex Low Binding Durapore (PVDF) syringefilter (Merk Millipore Ltd., Tullagreen, IE). VLPs were precipitated from the clarified supernatant by the addition of PEG-it Virus Precipitation Solution (System Biosciences, Palo Alto, CA, USA) at a ratio of 1:10. The supernatants were incubated at 4°C on a rotating shaker for 24-48 h prior to precipitation at 1500 x g for 30 min. The supernatant was carefully removed, and the VLP containing pellet was resuspended 0.05% - 0.1% of the initial volume with sterile PBS (pH 7.2). The resuspended pellets were kept at 4°C for short-term storage (1-3 weeks) or at -80°C for long-term storage.

### Nanoparticle tracking analysis

NTA was used to measure size and concentration of VLPs in different preparations. NTA measurements were performed using a NanoSight LM10 instrument (NanoSight, Amesbury, UK) consisting of a conventional optical microscope, high sensitivity sCMOS camera and a LM10 unit equipped with a 488 nm laser module. The samples were injected into the LM unit *via* the nanosight syringe pump with a constant flow rate of 50 µL/min with a 1 mL sterile syringe. Samples were diluted 1:5000. The capturing settings (shutter and gain) and analyzing settings were manually adjusted and kept constant between all samples that were recorded on the same day. NTA software (NTA 3.2 Dev Build 3.2.16) was used to capture three videos of 30 seconds and to analyze nanoparticle tracking data per sample.

## Results

### Development of hcAbs

**Figure 1:**
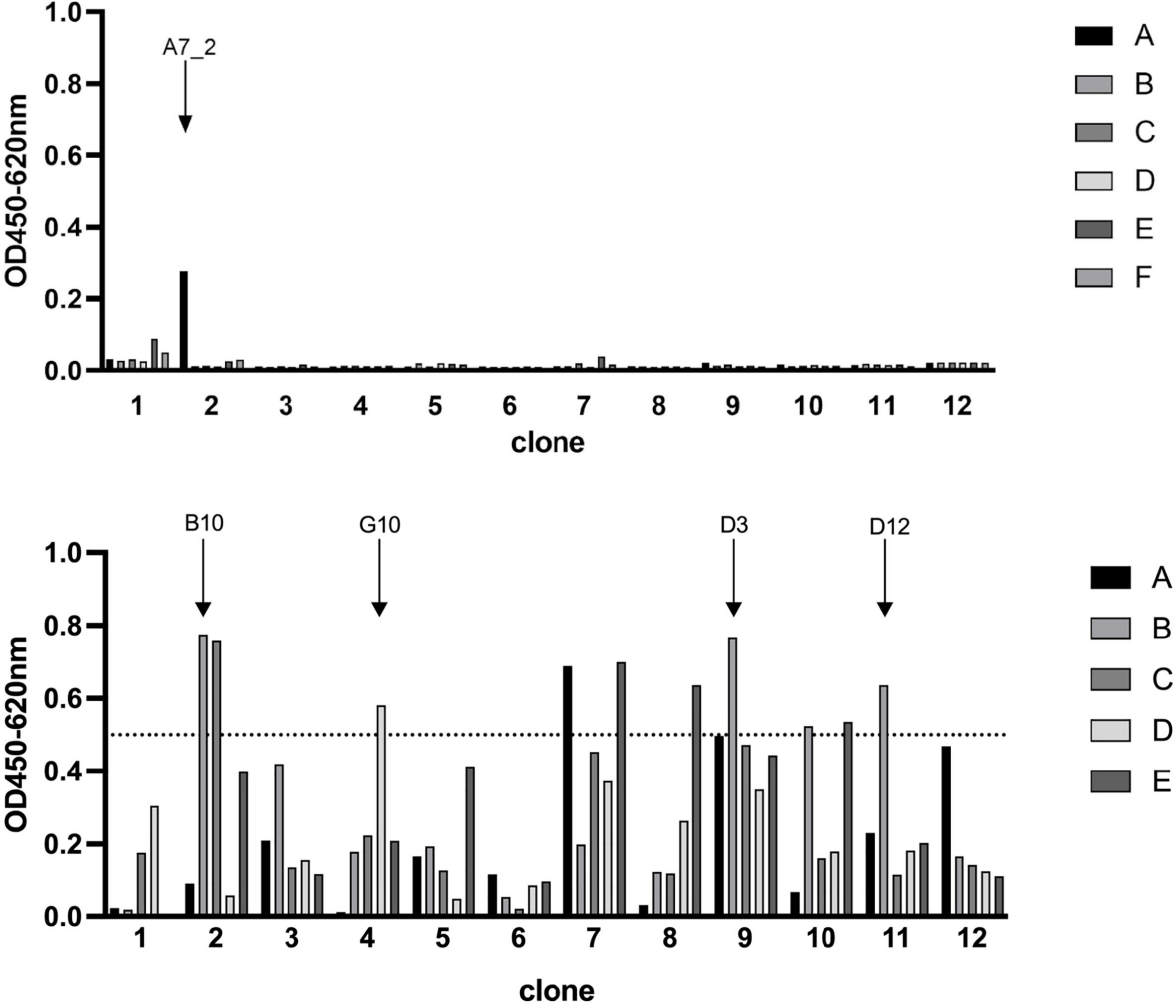
Phage display ELISA for the selection of SARS-CoV-2 Spike protein specific VHHs. Wells were coated with antigen (SARS-CoV-2 Spike protein, 3 µg/mL) and blocked with 100 µL/well PBS/1% casein. Detection of specific phages was performed with a HRP-conjugated M13 antibody (diluted 1:8000). Optical density was measured at 450 nm with a reference of 620 nm. Selected candidates are labeled with arrows. Blocking solution and a His-tagged irrelevant protein served as negative controls.

**Figure 2:**
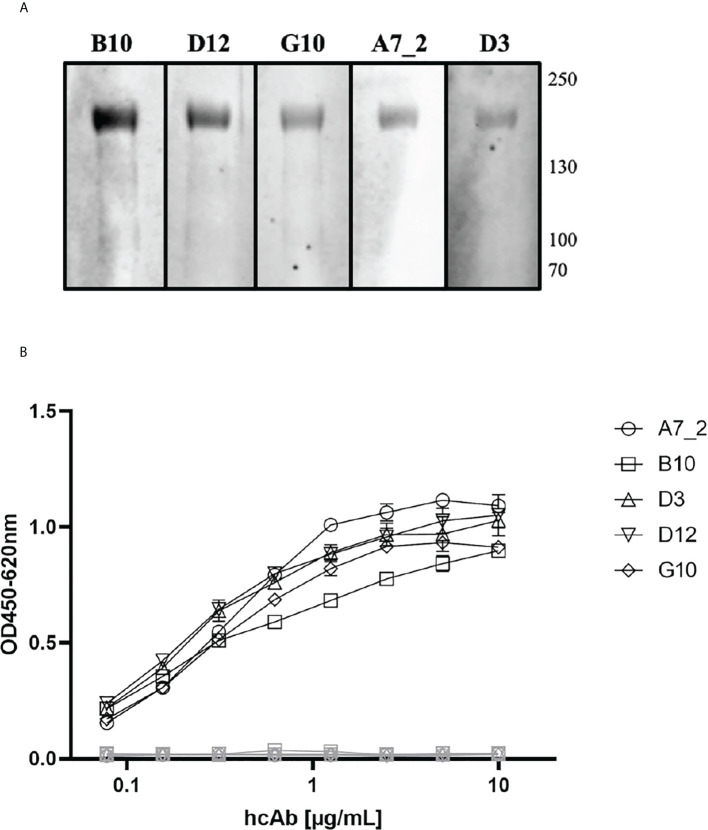
Characterization of SARS-CoV-2 Spike protein specific VHHs. **(A)** Western Blot analyses. Recombinant Spike protein was applied to a 4-12% SDS polyacrylamide gradient gel and blotted using the NuPage system. HcAb candidates (1 µg/mL) were incubated for 18 h. Detection took place using a HRP-conjugated secondary antibody and WesternBright Sirius as substrate solution. Human reconvalescent serum (1:100) served as positive control. Reference HIS-tagged protein (0.25 µg/mL) was used as negative control. **(B)** Indirect ELISA with recombinant SARS-CoV-2 Spike protein as antigen. Wells were coated with antigen (SARS-CoV-2 Spike protein, 3 µg/mL) and blocked with 100 µL/well PBS/1% casein. HcAbs were added in different dilutions. Detection was performed with a HRP-conjugated secondary antibody (ABIN1981272, 1 µg/mL) and optical density was measured at 450 nm with a reference wavelength of 620 nm. Blocking reagent and a His-tagged reference protein served as negative control. Mean and standard deviation of three independent measurements is shown.

Camelid single domain antibodies were selected by phage display out of a naïve camelid library. We could identify A7.2 as first candidate with a specific signal of 0.3 and around 10 more monophages with a specific ELISA signal of more than 0.5 when recombinant full length SARS-CoV-2 Spike protein was used for coating (**Figure 1**). The corresponding controls are summarized and shown in the **Supplementary File** as **Figure S2**. After sequencing, we chose 5 different candidates - A7.2 B10, D3, D12, and G10 - for recombinant expression of camelid heavy-chain-only IgG2 antibodies. Antibodies were purified by protein A chromatography (17) and characterized by Western blot analyses for their recognition of the recombinant SARS-CoV-2 Spike protein (**Figure 2A**). All hcAbs recognized the recombinant Spike protein in Western blot, whereas clone B10 showed a more intense signal than the other candidates.

### Characterization of hcAbs in different ELISA setups and SPR

The purified hcAbs were further characterized in different ELISA setups, to verify the optimal combinations and concentrations. First, the antibodies were serially diluted on recombinant SARS-CoV-2 Spike protein (**Figure 2B**) where they showed a recognition till a working concentration of 0.5 µg/mL. All hcAbs, including A7.2, showed a similar performance in this assay format.

**Figure 3:**
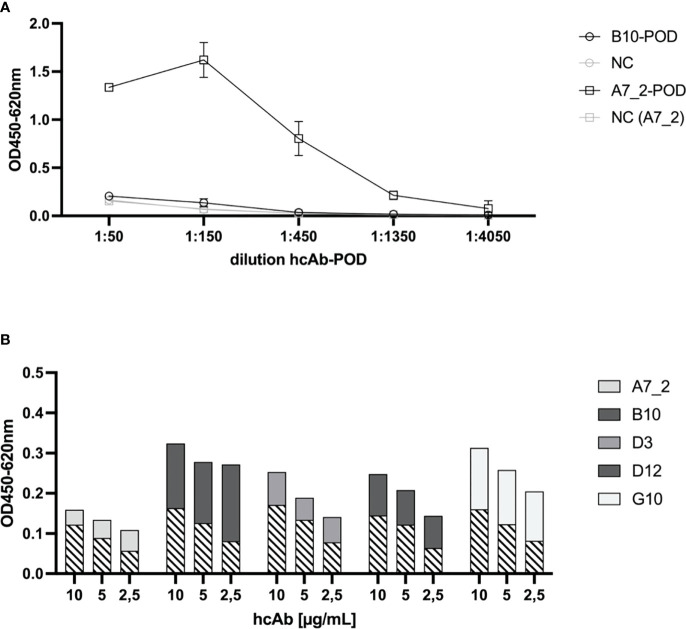
Sandwich Immunoassay setup for the detection of recombinant SARS-CoV-2 Spike protein. **(A)** HRP-conjugation of hcAbs B10 and A7.2. HcAbs B10 and A7.2 were conjugated with HRP to serve as secondary antibody in a sandwich ELISA. To detect the HRP conjugation, SARS-CoV-2 Spike protein was coated on the solid phase (1 µg/mL). HRP-conjugated hcAb B10 and A7.2 were added in different dilutions. Binding was detected by adding TMB substrate solution and optical density was measured at 450 nm with a reference wavelength of 620 nm. Mean and standard deviation of two independent measurements is shown. **(B)** Sandwich ELISA of hcAb candidates in different pairwise combinations. Purified hcAbs were coated on the solid phase in different concentrations of 10, 5 and 2.5 µg/mL. SARS-CoV-2 Spike protein was added (2.5 µg/mL). HcAb A7.2 was applied as HRP-conjugated secondary antibody in a dilution of 1:1600. Detection of optical density was performed at 450 nm with a reference wavelength of 620 nm. Cross-hatched areas represent the background value for negative control (coated blocking solution instead of hcAb).

In the next step, we tested pairwise combinations to find the optimal catcher and detector antibodies for a sandwich immunoassay principle (**Figure 3**). To ensure a direct detection, we conjugated hcAb B10 and A7.2 to HRP and tested their performance in a direct ELISA (**Figure 3A**). A7.2 showed a more succinct labeling with HRP and a better performance in the assay compared to B10. Therefore, we used HRP-conjugated hcAb A7.2 in a dilution of 1:1600 as detection antibody. **Figure 3B** shows the results for the sandwich immunoassay. We coated all hcAb candidates in different concentrations of 10, 5 and 2.5 µg/mL on the solid phase. Recombinant SARS-CoV-2 Spike protein was used as antigen and HRP-conjugated hcAb A7.2 as detection antibody. The usage of B10 and G10 as catcher hcAb and HRP-conjugated hcAb A7.2 as detector seemed to be the most sensitive combination in this sandwich immunoassay format (**Figure 3B**, 2nd). Nevertheless, we saw a linear decrease from 10 to 2.5 µg/mL for G10. HcAb B10 showed a non-linear decrease which implicates that the used concentrations are still in the saturated level and can be further diluted. For ongoing experiments we therefore decided to use hcAb B10 and A7.2 as lead candidates for further characterizations.

**Figure 4:**
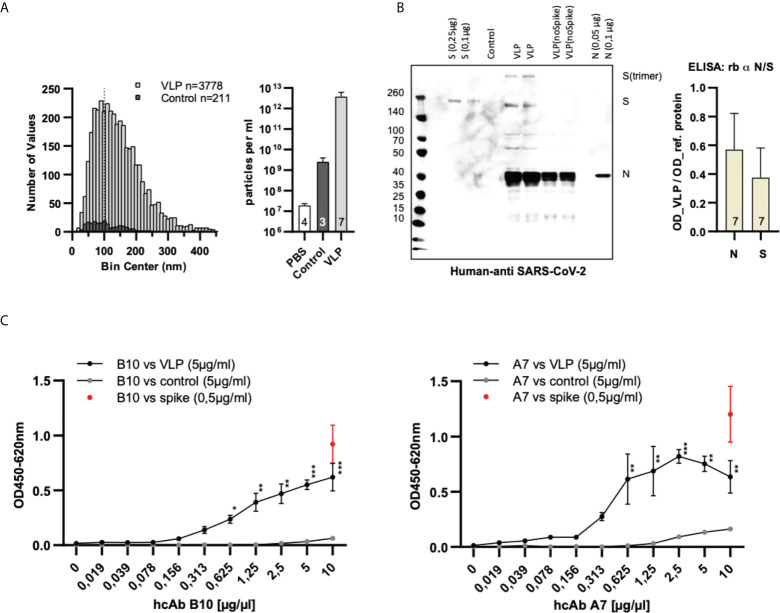
Characterization and specific binding of SARS-CoV-2 VLPs. **(A)** Nanoparticle tracking analysis of particle size and concentration. VLPs were purified from cell culture supernatants transfected with expression plasmids of four SARS-CoV-2 structural proteins (S, N, M and E). Data are derived from seven independent transfections (VLPs) or three untransfected cell culture supernatants (control). Samples were diluted 1:5000 prior to measuring in NanoSight LM10. The size distribution of individual particles and the particle concentration of the samples is shown. The dotted line at 100 nm indicates the approximate size of authentic SARS-CoV-2 VLPs (23). **(B)** Confirmation of the presence of Spike- and nucleoprotein in SARS-CoV-2 VLPs. For the detection of Spike- and nucleoprotein on SARS-CoV-2 VLPs human reconvalescent serum was used in a dilution of 1:100 in western blot anlyses. Reference proteins served as controls. For the ELISA, VLPs (5 µg/mL) were coated on the solid phase and detected with commercial antibodies specific for the Spike- and the nucleoprotein. Reference proteins were used in a concentration of 0.25 µg/mL to serve as control. Mean values and standard deviation of seven independent VLP preparations is shown. **(C)** Dose-dependent detection of SARS-CoV-2 VLPs using hcAbs B10 and A7.2. VLPs, control particles or Spike protein were coated on the solid phase. HcAbs B10 and A7.2 were applied in different dilutions. Detection was performed using a HRP-conjugated secondary antibody. The optical density was measured at 450 nm with a reference wavelength of 620 nm. Mean and standard deviation of three independent measurements is shown. ELISA: 2-log dilution of B10 and A7.2. Kruskal Wallis test with Benjamini, Krieger and Yekuteli correction for multiple comparison against 0 µg/ml *** p<.001, ** p<.01, * P<.05. n=3.

In **Figure 4** we have summarized the results for our generated SARS-CoV-2 VLPs. We could prove that the generated VLPs have the expected particle size (**Figure 4A**) and carry the Spike (S) as well as the nucleoprotein (N) which were detectable with human reconvalescent patient sera in Western blot experiments (**Figure 4B**). The presence of E and M protein was not specifically tested because their presence is necessary for the release of VLPs from the host cell (24, 25). The VLPs were used as antigen coated on the solid phase and detected by either hcAb B10 or A7.2 followed by a HRP-conjugated secondary antibody (**Figure 4C**). The dilution series revealed a working concentration of 1.25 µg/mL for hcAb B10 and 0.6 µg/mL for hcAb A7.2.

Next, we characterized the hcAb candidate B10 in surface plasmon resonance spectrometry (SPR). SPR measurements were performed with immobilized SARS-CoV-2 Spike protein (RBD domain) recombinantly expressed with a murine Fc part (S prot RBDmFc). The immobilization level of the ligand was 24.1 RU. According to the formula given in the experimental section and the molecular weight of the ligand and the analyte, the R_max theoretical_ can be calculated as follows:


$$\frac{24.1\;\text{RU}(\text{S prot RBDmFc dimer})}{104\;\text{kDa}(\text{S prot RBDmFc dimer})}\times 79.5\;\text{kDa B}10\text{ dimer}=18.4\;{\text{R}}_{\text{max theoretical}}$$

$$\frac{16.2\;\text{Rmax analysis}}{18.4\;\text{Rmax theoretical}}=0.9_{stoichiometry}$$

These results show a stoichiometry of 1:0.9 which implies that one molecule hcAb B10 bound to one molecule of S prot RBDmFc.

**Figure 5:**
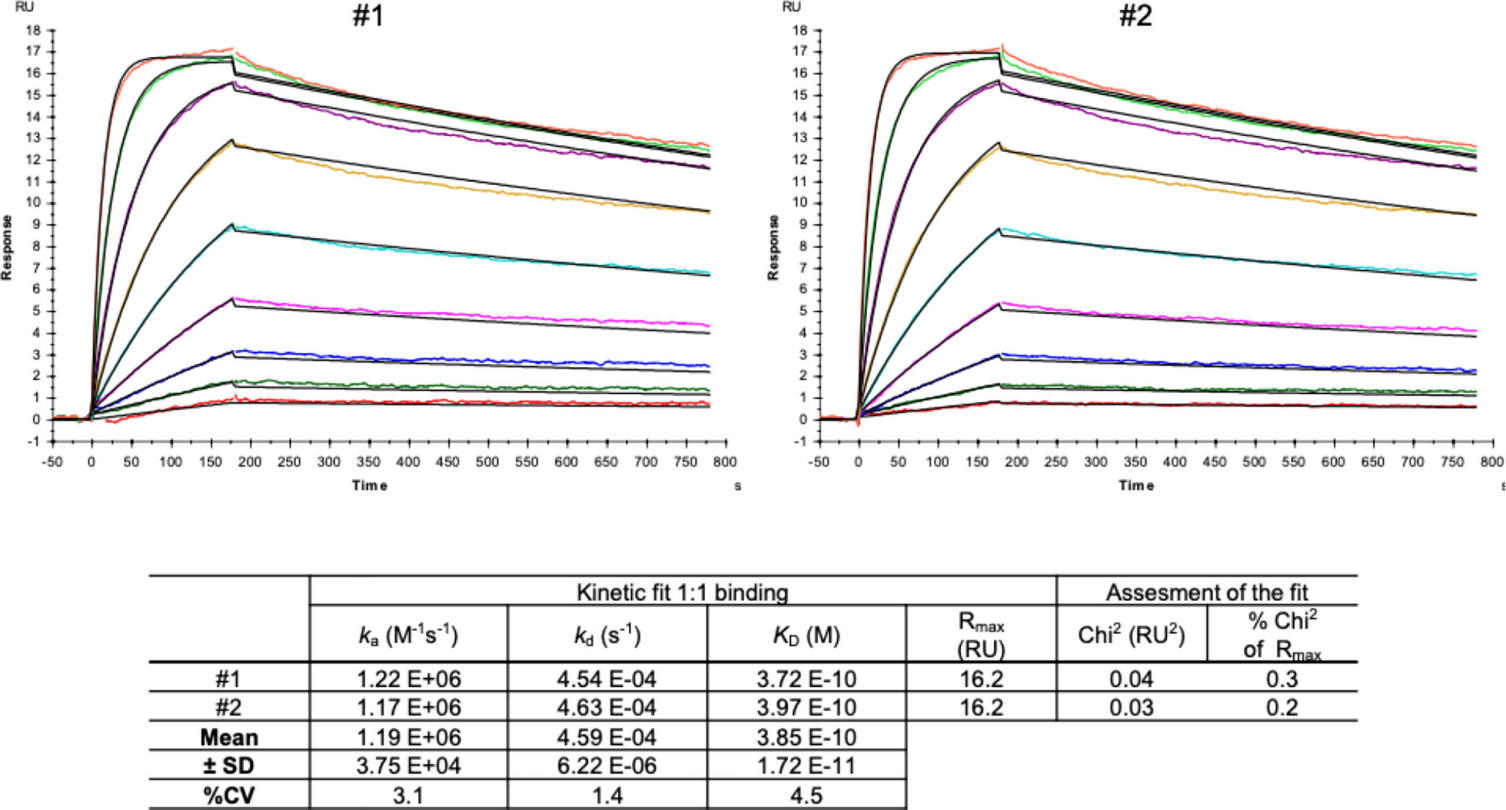
SPR measurements of hcAb B10. SARS-CoV-2 Spike protein-RBD-mFc (Acrobiosystem; SPD-C5259) was used as antigen and captured by a covalently immobilized anti-IgG1 antibody on a C1 sensor chip. Increasing concentrations of hcAb B10 (0.23 - 60 nM) were injected. Analyte responses were corrected for unspecific binding and buffer responses. The sensograms were fitted with a monovalent binding model using Biacore evaluation software.

The Kd value for hcAb B10 could be measured reproducible as shown in **Figure 5** and gives a value of 0.39 nM (**Figure 5**). The very low immobilization of the ligand (24.1 RU) reduces the avidity effects given by the bivalent nature of antibody molecules and allows an analysis in a 1:1 binding model (19, 20). Further, flat sensor chips without dextran were used to minimize sterical accessibility and reduce bivalent binding events (26).

### Neutralization capacity of hcAbs

**Figure 6:**
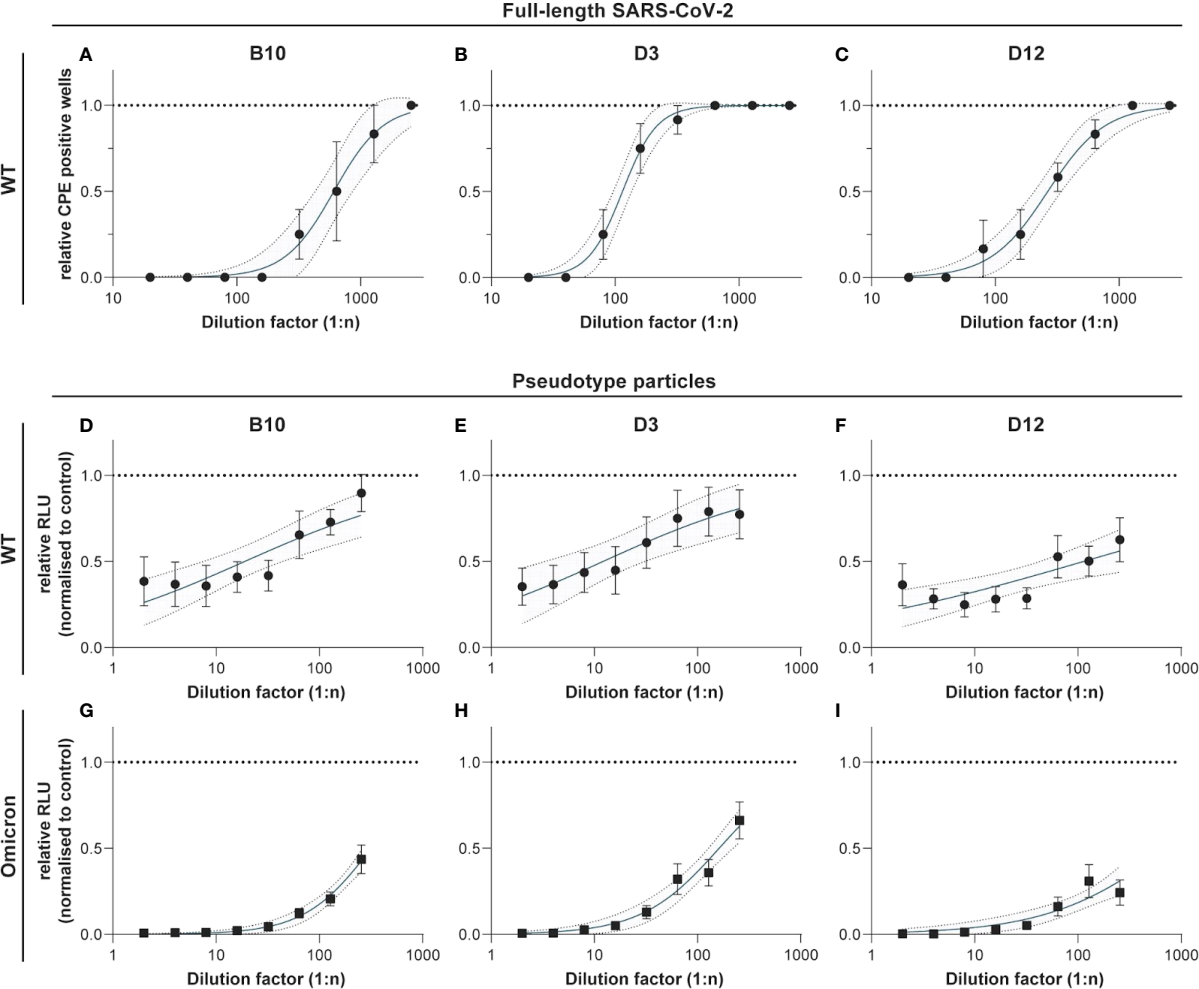
Neutralization experiments with SARS-CoV-2 wildtype virus and pseudotyped viruses (wildtype and Omicron). HcAbs B10, D3 and D12 were used for neutralization experiments with the wildtype virus hCoV-19/Germany/BY-Bochum-1/2020 (B.1.1.70) **(A–C)** and with a propagation-incompetent VSV*ΔG pseudovirus system for the wildtype **(D–F)** and Omicron **(G–I)**. VSV*ΔG were incubated with a two-fold dilution of hcAbs and used for infection of VeroE6 cells. Neutralization was measured using a Firefly luciferase (FLuc) reporter system. The reciprocal antibody dilution causing 50% inhibition of the luciferase reporter was calculated (ND50) in 3 independent experiments. Mean and standard deviation are shown as well as a 95% confidence interval set off with grey.

Neutralization assays were performed with full length SARS-CoV-2 wildtype and SARS-CoV-2 pseudotyped viruses for the wildtype, and the Omicron variant, respectively. **Figure 6** shows the results for the neutralization capacity of hcAb B10, D3, and D12 for the wildtype virus (A-C), and the pseudotyped viruses of wildtype (D-F) and Omicron (G-I). B10 and D12 were strongly neutralizing the full length wildtype virus at a concentration of 1 µg/mL. D3 showed an intermediate neutralizing activity between 4.3 and 5.8 µg/mL. In neutralization assays with the pseudotype particles (**Figure 6D–I**) the hcAbs showed a similar capacity as shown for the full length wildtype virus. We found that also the new variant of concern B1.1.529 (Omicron) was neutralized by the hcAbs B10, D3 and D12 in a range of 0.1 - 0.35 ng/mL (ND50). For hcAb A7.2 there was no neutralization detected (data not shown).

## Discussion

Camelid heavy-chain-only antibodies represent a promising antibody format for therapeutic as well as *in vitro* diagnostic applications. These types of antibodies provide favourable properties such as a high thermostability and an improved binding behaviour compared to conventional antibody formats (27). The antigen-binding moiety of those antibodies, so called nanobodies, serve as one of the smallest intact antigen binding unit (15 kDa) and are used mainly for therapeutic applications (28). For standard *in vitro* diagnostic assay systems, Fc tagged antibodies are widely used to build up a sensitive detection. Current *in vitro* diagnostic systems use polyclonal or monoclonal full length antibodies as detector antibodies coupled to HRP or biotin to provide a measurable and sensitive detection of the target. So far, there are several assay systems based on camelid nanobodies published (29–34), where nanobodies were coupled to HRP, alkaline phosphatase or beads but none of these are using camelid heavy-chain-only antibodies for detection. This study identifies suitable camelid heavy-chain-only antibody candidates specific for SARS-CoV-2 Spike protein selected by phage display from a camelid naïve library. We have expressed the nanobodies as heavy-chain-only antibody formats of an IgG2 isotype to establish tools for a standardized ELISA based detection system.

Normally, when using naïve libraries lower affinities are expected because the immune system was not specifically challenged against the target (35). In addition, the size of our library was with 1-3x10^7^ PBMCs per donor animal (3 in total) very small but the five candidates chosen from the selection rounds in this study showed good binding properties against the target. For the construction of the library, from 55 picked clones only 4 clones had no VHH insert (7.3%). From 20 sequenced clones, all had a proper VHH insert in the open reading frame. It is published that also potent antigen-binding domain binders can be recovered from relatively small naïve libraries (36). When using the pADL vector for phage display, we observed a high rate of self ligation. An alternative vector is pCOMB from Barbas et al. which is commonly used with a better ratio (15).

**Figure 7:**
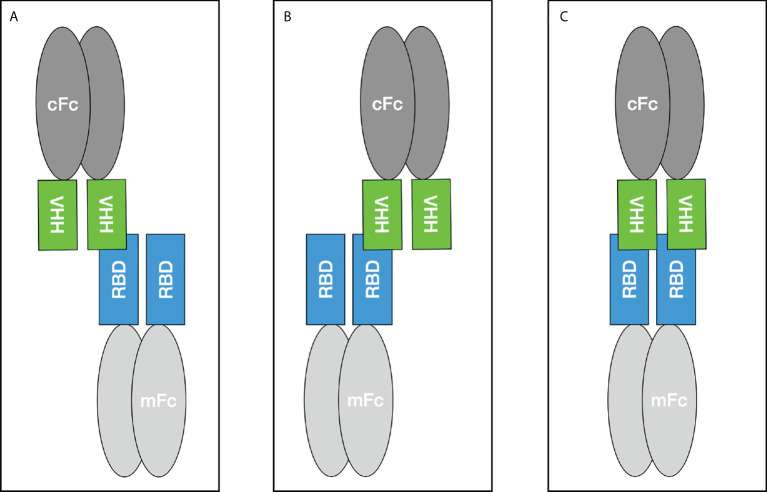
Binding scenarios for the Fc-tagged VHH construct and the SARS-CoV-2 RBD domain fused to a murine Fc tag during SPR measurements. mFc - murine Fc, cFc - camelid Fc.

During the biopanning, several screening rounds were performed to avoid avidity effects caused by the fact that up to 5 VHHs can be presented on one phage (37). After the phage ELISAs, which served purely as Yes/No answer, the selected VHHs were expressed as monomer and reconstituted as camelid heavy-chain-only antibodies to validate the specific binding to the target. Five candidates were expressed and further characterized with Western Blot and different ELISA setups. According to the results hcAb B10 and HRP-labeled A7.2 were chosen as capture and detection antibody in the sandwich ELISA. Due to the performance it was possible to reduce the coating concentration for hcAb to less than 1 µg/mL. Together with HRP-labeled hcAb A7.2 as a detector antibody, a very potent antibody pair could be established. The successful conjugation of hcAb A7.2 with a routinely used enzyme such as HRP offers a standardized use of hcAbs also for *in vitro* diagnostic applications. Unfortunately, for hcAb B10 the labeling with HRP was less efficient than for A7.2. This could be due to an unspecific labeling within the antigen-binding domain and needs to be further optimized. The hcAb B10 was characterized in SPR with an affinity constant of 0.39 nM in a monovalent binding model which is compared to other SARS-CoV-2 nanobodies from immune or synthetic libraries, a highly affine binder. Due to the reconstitution of the VHH with a camelid IgG2 Fc region there might be avidity effects because of the bivalent structure of the hcAb and the dimeric form of the RBD fused to a murine Fc which was used for SPR measurements. To avoid this problem, a sensor chip without dextran was used because the high flexibility of dextran matrices leads to a higher percentage of bivalent bindings (26). Further, the antigen was immobilized in a very low concentration of 24.1 RU as described previously to eliminate avidity effects and allow analysis in a 1:1 binding model (19, 20). The calculation of R_max theoretical_ gives a value of 0.9 for the stoichiometry and proves the binding in a 1:1 model where one molecule hcAb B10 bounds to one molecule RBDmFc. To make it visual, three different scenarios result from this interaction as shown in **Figure 7**. The binding in scenario A and B are of a monovalent character. Scenario C is dependent on the sterical conditions and the epitope of the VHH. However, our results prove the feasibility of assay systems completely designed and coordinated with camelid heavy-chain-only antibodies. These opportunities offer new opportunities and provide flexible tools for future applications.

Further, we could show that these hcAb candidates are not only useful as *in vitro* diagnostic tests components. The neutralization data for the full length wildtype virus as well as the pseudotyped wildtype and the new variant of concern B1.1.529 (Omicron) reveal a possible therapeutic application too. With four out of five candidates which are able to effectively neutralize the SARS-CoV-2 wildtype virus as well as the newly emerged Omicron variant we can provide highly efficient tools useful for a range of different applications. With regard to other SARS-CoV-2 variants it is necessary to validate the performance of all camelid candidates, their binding efficacy to the RBD and wether their binding is overlapping with ACE binding. In addition, to solve the structure of the hcAbs in complex with the Spike protein and to gain more insights into the mechanism of neutralization, cryo-electron microscopy will be a beneficial approach. For camelid antibodies it is described that all CDR regions can potentially make contact with the antigen (38). To reconstitute how our hcAbs recognize the RBD and induce neutralisation is an upcoming project.

As recently described, the therapeutic potential of current human antibodies against the newly emerged Omicron variant is reduced caused by the increased number of mutations in the RBD domain and the resulting loss of specificity (39). Our hcAb portfolio can therefore provide an alternative and improve the current availability of antibodies for both, a diagnostic as well as a therapeutic use.

## Conclusion

Camelid antibodies have a high potential for diagnostic and therapeutic applications because of their exclusive properties compared to routinely used human or mouse antibodies. The approval of the first camelid therapeutic antibody was an important step which paved the way for this next generation antibody formats. Due to the excellent properties of camelid antibodies such as a high safety and stability small sized VHH but also heavy-chain-only antibodies represent benefits for the development of novel assays and treatments. Especially, for *in vitro* diagnostic assay systems camelid antibodies provide substantial advantages. They can easily be adapted to a routine detection by using corresponding Fc parts and specific secondary antibodies. To create novel tools and establish applications based on camelid antibodies will significantly impact future developments in the antibody field. The antibodies described in this study will be validated further for their potential in diagnostic applications but also with regard to their neutralizing capacity of upcoming SARS-CoV-2 variants to provide efficient tools in COVID-19 related diagnostics and therapy.

## Data availability statement

The original contributions presented in the study are included in the article/**Supplementary Material**. Further inquiries can be directed to the corresponding author.

## Ethics statement

The animal study was reviewed and approved by Ministry of Environment, Health and Consumer Protection.

## Author contributions

AS designed the phage display experiments and performed the panning rounds, hit selection, recombinant expression, purification and ELISA characterizations of the hcAbs. SH developed the protocols for VLP expression and characterization and preformed the Western Blot experiments. GBA contributed to the production and characterization of VLPs. TLM and PA prepared and performed the neutralization experiments. SPf, MH, and SPö coordinated the neutralization experiments and provided the Omicron plasmid. OKE, JK-M and KH initiated the project, coordinated and supervised the experiments. All authors contributed to the writing of the manuscript and agreed to the final version of it.

## Acknowledgments

We would like to thank Dr. Michael Zenn from Biaffin GmbH & Co.KG for performing the SPR measurements of hcAb B10 and the data analysis support. In addition we would like to thank Elena Vidal Blanco of the Department for Medical and Molecular Virology for technical support. We thank Gert Zimmer, Institute for Virology and Immunology, Switzerland and Department of Infectious Diseases and Pathobiology (DIP), Vetsuisse Faculty, University of Bern, Switzerland for providing plasmids and reagents. Funded by the Deutsche Forschungsgemeinschaft (DFG, German Research Foundation) – Projektnummer 491466077.

## Conflict of interest

KH is shareholder and CEO of new/era/mabs GmbH. AS is employed at new/era/mabs. OE is associated with Wimedko GmbH. GBA is employed at Wimedko GmbH. JK-M was employed at Wimedko GmbH. KH and AS are inventors of the European patent application No. EP21212985.2.

The remaining authors declare that the research was conducted in the absence of any commercial or financial relationships that could be construed as a potential conflict of interest.

## Publisher’s note

All claims expressed in this article are solely those of the authors and do not necessarily represent those of their affiliated organizations, or those of the publisher, the editors and the reviewers. Any product that may be evaluated in this article, or claim that may be made by its manufacturer, is not guaranteed or endorsed by the publisher.
