# Supplementary figures and images for: SARS-CoV-2 neutralizing camelid heavy-chain-only antibodies as powerful tools for diagnostic and therapeutic applications
Source: Front Immunol. 2022 Sep 14;13:930975. doi: 10.3389/fimmu.2022.930975 (PMC9517167; doi:10.3389/fimmu.2022.930975)

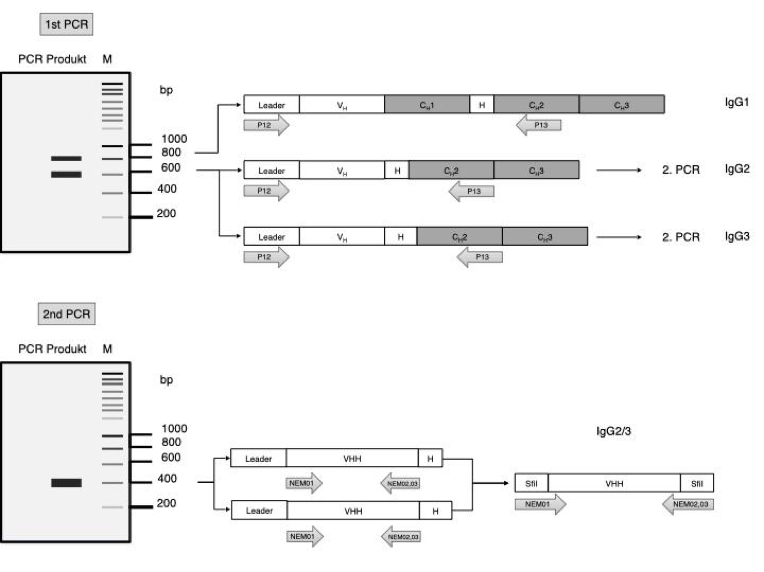

Supplement: Supplementary file 1 [file Image_1.tiff]

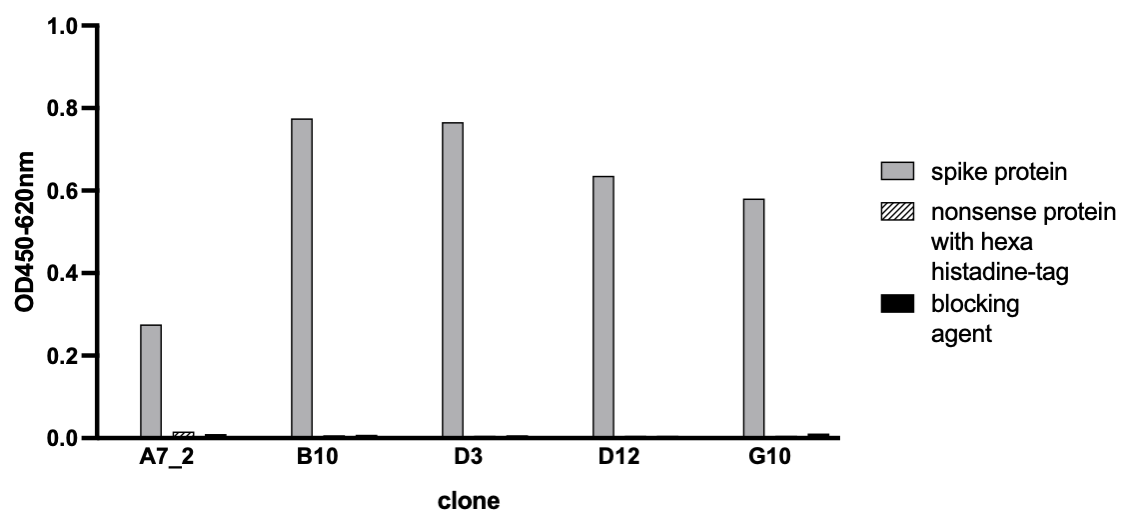

Supplement: Supplementary file 2 [file Image_2.tiff]
